# Red Blood Cells and Endothelium Derived Circulating Extracellular Vesicles in Health and Chronic Heart Failure: A Focus on Phosphatidylserine Dynamics in Vesiculation

**DOI:** 10.3390/ijms241411824

**Published:** 2023-07-23

**Authors:** Rosa Suades, Alba Vilella-Figuerola, Teresa Padró, Sonia Mirabet, Lina Badimon

**Affiliations:** 1 Cardiovascular Program ICCC, Research Institute of Hospital Santa Creu i Sant Pau, IIB Sant Pau, 08049 Barcelona, Spain; rsuades@santpau.cat (R.S.); avilella@santpau.cat (A.V.-F.); tpadro@santpau.cat (T.P.); 2 Centro de Investigación Biomédica en Red en Enfermedades Cardiovasculares (CIBERCV), Instituto de Salud Carlos III (ISCIII), 28029 Madrid, Spain; 3 Cardiology Department, Hospital Santa Creu i Sant Pau, 08025 Barcelona, Spain; smirabet@santpau.cat; 4 Cardiovascular Research Chair, Universitat Autònoma de Barcelona (UAB), 08193 Barcelona, Spain

**Keywords:** annexin V, connexin 43, endothelial cells, extracellular vesicles, heart failure, leukocytes, microvesicles, phosphatidylserine, platelets, red blood cells

## Abstract

Circulating extracellular microvesicles (cEVs) are characterised by presenting surface antigens of parental cells. Since their biogenesis involves the translocation of phosphatidylserine (PS) from the inner to the outer leaflet of the plasma membrane, exposed PS has been considered as a recognition hallmark of cEVs. However, not all cEVs externalise PS. In this study, we have phenotypically and quantitatively characterised cEVs by flow cytometry, paying special attention to the proportions of PS in chronic heart failure patients (cHF; *n* = 119) and a reference non-HF group (*n* = 21). PS^−^-cEVs were predominantly found in both groups. Parental markers showed differential pattern depending on the PS exposure. Endothelium-derived and connexin 43-rich cEVs were mainly PS^−^-cEVs and significantly increased in cHF. On the contrary, platelet-derived cEVs were mostly PS^+^ and were increased in the non-HF group. We observed similar levels of PS^+^- and PS^−^-cEVs in non-HF subjects when analysing immune cell-derived Evs, but there was a subset-specific difference in cHF patients. Indeed, those cEVs carrying CD45^+^, CD29^+^, CD11b^+^, and CD15^+^ were mainly PS^+^-cEVs, while those carrying CD14^+^, CD3^+^, and CD56^+^ were mainly PS^−^-cEVs. In conclusion, endothelial and red blood cells are stressed in cHF patients, as detected by a high shedding of cEVs. Despite PS^+^-cEVs and PS^−^-cEVs representing two distinct cEV populations, their release and potential function as both biomarkers and shuttles for cell communication seem unrelated to their PS content.

## 1. Introduction

Circulating extracellular vesicles (cEVs) are a heterogeneous population of small membrane particles involved in several physiological processes such as cell adhesion, cell–cell crosstalk, haemostasis, and thrombosis, and it has been demonstrated that they have a role in disease [1,2,3]. Microvesicles, with a size range between 20 and 1000 nm and originating by cellular plasma membrane blebbing [4,5], are characterised by presenting, in their membrane, antigens from their parental cells and by carrying active molecules such as proteins, metabolites, RNA, or DNA [4,5,6]. Until very recently, it was universally thought that cEV release always involves the loss of lipidic asymmetry of the plasma membrane, exposing phospholipid phosphatidylserine (PS) in the outer membrane of the vesicle [5] being PS a common feature of all cEVs. However, we now know that there are also cells that release cEVs without PS externalization [7,8,9,10,11,12].

Affecting approximately 2% of the worldwide population over 65 years of age and with an increasing incidence, heart failure (HF) accounts for a high proportion of hospitalization and cardiovascular death around the globe [13]. It is characterised by an impairment of the cardiac function that results in the inability of the cardiac muscle to reach the body’s metabolic demands. This complex clinical condition can derive from genetic or environmental insults that induce significant alterations of the heart function (decreased cardiac output, systolic or diastolic dysfunction) and structure (left ventricle dilation or hypertrophy, maladaptive remodelling, fibrosis) [14,15].

Accumulating evidence suggests that EVs participate in the pathogenesis of HF development, exerting both protective [16] and detrimental [17] effects. Several studies conducted over the last years have focused on the direct association between heart failure and EVs of endothelial origin [18,19], even in HF patients with other comorbidities such as metabolic syndrome [20,21], subclinical hypothyroidism [22], and insulin resistance in non-diabetics [23]. In this regard, a dysregulated signature of CD31^+^ endothelial-derived EVs could predict chronic HF phenotypes, improving conventional HF markers [24] and assess the risk of three-year cumulative cardiovascular events [25]. We previously investigated white cell-derived EVs [26] and platelet-derived EVs (pEVs) [12] in the circulation of chronic heart failure (cHF) patients. pEVs were also found to potentially contribute to a HF-related hypercoagulable state [27].

Although we [12] and others [28] reported that patients with chronic conditions such as cHF present higher numbers of PS^+^-pEVs, the role of phosphatidylserine externalization in EVs remains unclear. Therefore, here, we aimed to study cEVs with and without PS membrane exposure (PS^+^-pEVs and PS^−^-cEVs) in patients with cHF and investigated their parental blood and vascular cell origin.

## 2. Results

### 2.1. Circulating Extracellular Vesicles with and without Phosphatidylserine Membrane Exposure

The distribution of global PS^−^-EVs differed between the non-HF and cHF groups, as shown in Table 1. In the reference non-HF group, the total number of EVs in circulation was 804 (301–1105)/µL, with 51% being PS^+^-EVs and 49% being PS^−^-EVs. In the cHF group, the total number of cEVs was 1079 (426–4702)/µL, and of those, 16% were PS^+^-EVs and 84% were PS^−^-EVs, indicating a differential exposure of PS (annexin V [AV]^+^ and AV^−^) in cEV membranes in non-HF individuals and cHF patients (*p* < 0.001, both), as well as between both groups (*p* = 0.022 (for PS^+^-EVs) and *p* = 0.007 (for PS^—^EVs), respectively).

The relative amount of cEVs from a distinct cell origin (Table 1) reveals that the majority of PS^+^-EVs were platelet-derived EVs (CD41a^+^-pEVs (integrin α_IIb_β_3_)), accounting for 86% in the reference non-HF group and 53% in the cHF patient group (*p* < 0.001). Platelets also released PS^−^-cEVs with a 20% frequency in the non-HF subjects and a 7% frequency in the cHF patients (*p* = 0.032). In contrast, endothelial-derived EVs (CD309^+^-eEVs; vascular endothelial growth factor receptor 2 (VEGFR2)) without PS membrane exposure (PS^−^) occurred with a frequency of 13% in the non-HF and 23% in the cHF (*p* = 0.012) subjects, while levels of CD309^+^/AV^+^-eEVs were almost null both in the non-HF and cHF groups. Leukocytes are also a source of both PS^−^-EVs (CD45^+^-LEVs; leukocyte common antigen [LCA]; *p* = 0.586), had a 6.01% and 5.81% frequency in the non-HF individuals and cHF patients, respectively, and PS^+^-CD45^+^-LEVs, represented 9.01% of EVs in the non-HF controls and 16.49% of cEVs in patients with cHF (*p* = 0.016). Other identified cell-derived EVs were from erythrocytes (CD235ab^+^-ErEVs [glycophorin a and b]). While similarly high levels of PS^−^-ErEVs were found in both groups (27.4% in non-HF and 19.56% in cHF; *p* = 0.245), PS^+^-ErEVs were mainly found in cHF patients (24%), representing only 1.33% in non-HF subjects. Finally, an important proportion of connexin-43-carrying EVs (CX43^+^-EVs) were AV^−^ (18.06% and 27.2% in non-HF controls and cHF patients, respectively; *p* = 0.028). A small amount of CX43^+^/AV^+^-EVs was detected (0.56% in non-HF versus 1.88% in cHF group; *p* = 0.001). In summary, the circulating PS^−^-cEVs with a different cellular origin and relative releases differed between the cHF and the reference non-HF groups.

### 2.2. Differential Patterns between Phosphatidylserine Positive and Negative Circulating Extracellular Vesicles in Non-Heart Failure Reference Group and Patients with Chronic Heart Failure

#### 2.2.1. Endothelial-Derived Circulating Extracellular Vesicles

**Figure 1:**
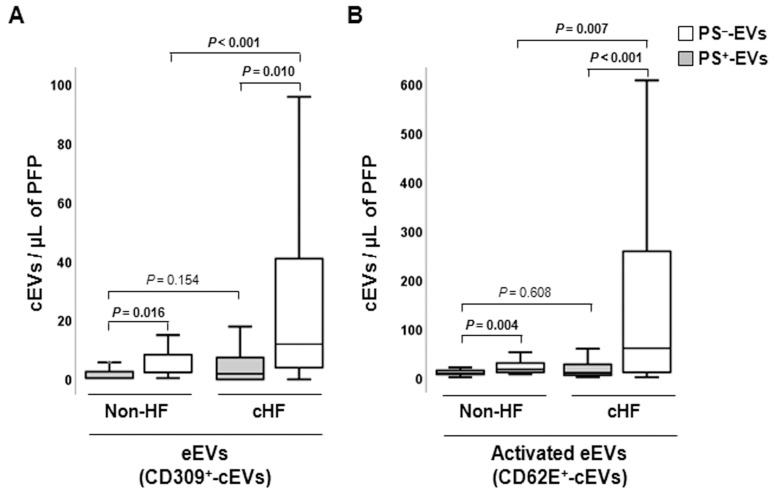
Distribution of circulating extracellular vesicles of endothelial origin in reference non-heart failure and chronic heart failure groups. Box and whisker plots show numbers of cEVs per microliter of platelet-free plasma (cEVs/µL of PFP) from (**A**) non- (CD309^+^) and (**B**) activated (CD62E^+^) endothelial cells in non-heart failure control subjects (*n* = 21) and patients with chronic heart failure (*n* = 119). Lines within boxes represent median values, the upper and lower boxes represent the 25th and 75th percentiles, respectively, and the upper and lower boxes outside the boxes represent the 10th and 90th percentiles, respectively. A *p* < 0.05 was considered significant (Mann–Whitney U test). *p*-values in bold correspond to significant differences. CD indicates cluster of differentiation; cHF, chronic heart failure; cEVs, circulating extracellular vesicles; eEVs, endothelial-derived EVs; HF, heart failure; PFP, platelet-free plasma; PS^+^, EVs exposing phosphatidylserine; and PS^−^, EVs that do not expose phosphatidylserine.

Differential distributions of PS^+^- and PS^−^ endothelial-derived cEVs were observed, with PS^−^-EVs accounting for the major proportion of eEVs both in the cHF and non-HF groups (*p* ≤ 0.016, all), as displayed in Figure 1. The endothelium-originated PS^−^-eEV levels were higher in cHF patients compared to non-HF subjects, for both eEVs presenting markers of non-activated (CD309^+^/AV^−^ (VEGFR2); *p* < 0.001) and activated (CD62E^+^/AV^−^ (E-selectin); *p* = 0.007) endothelial cells. The PS^+^-eEV levels were similar in both non-HF and cHF blood (non-significant differences), independently of the cell activation status.

#### 2.2.2. Erythrocyte-Derived Circulating Extracellular Vesicles

**Figure 2:**
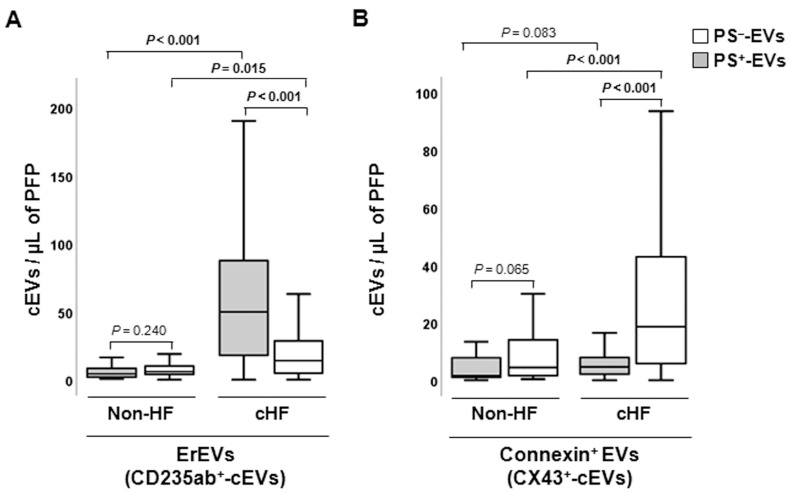
Distribution of red blood cell- and connexin 43-rich circulating extracellular vesicles in reference non-heart failure and chronic heart failure groups. Box and whisker plots show numbers of cEVs per microliter of platelet-free plasma (cEVs/µL of PFP) from (**A**) erythrocytes (CD235ab^+^) and (**B**) carrying connexin-43 (CX43^+^) in non-heart failure control subjects (*n* = 21) and patients with chronic heart failure (*n* = 119). Lines within boxes represent median values, the upper and lower boxes represent the 25th and 75th percentiles, respectively, and the upper and lower boxes outside the boxes represent the 10th and 90th percentiles, respectively. A *p* < 0.05 was considered significant (Mann–Whitney U test). *p*-values in bold correspond to significant differences. CD indicates cluster of differentiation; cHF, chronic heart failure; cEVs, circulating extracellular microvesicles; ErEVs, erythrocyte-derived EVs; HF, heart failure; PFP, platelet-free plasma; PS^+^, EVs exposing phosphatidylserine; and PS^−^, EVs that do not expose phosphatidylserine.

Erythrocyte-derived cEVs were increased in patients with cHF compared with non-HF individuals, independently of phosphatidylserine exposure (PS^−^-EVs and PS^+^-EVs, *p* ≤ 0.015, both), as shown in Figure 2A. In the cHF patients, significantly higher proportions of CD235ab^+^/AV^+^-EVs were observed compared to CD235ab^+^/AV^−^-EVs (*p* < 0.001), while no differences between them were observed in the reference non-HF group (*p* = 0.240).

#### 2.2.3. Connexin-43-Carrying Circulating Extracellular Vesicles

Increased levels of PS^−^-EVs carrying connexin-43 (CX43^+^/AV^−^-EVs) were observed in the cHF patients in comparison with the non-HF subjects (*p* < 0.001), while CX43^+^-PS^+^-EVs were found in comparable proportions in non-HF controls and cHF patients (*p* = 0.083). Despite both groups showing higher proportions of connexin-43-carrying PS^−^-EVs than PS^+^-EVs, only in the cHF patient group was this rise significant (*p* < 0.001). No differences between CX43^+^/AV^+^ and CX43^+^/AV^−^ were observed in the non-HF group (*p* = 0.065) (Figure 2B).

#### 2.2.4. Platelet and Inflammatory Cell-Derived Circulating Extracellular Vesicles

Platelet-derived cEVs were mainly PS^+^-EVs in both the non-HF subjects and patients with cHF, as previously shown [12]. In addition, while PS^+^-pEVs were found in lower amounts in cHF compared to non-HF controls, there were no differences in cEVs that did not expose PS, with the exception of CD31^+^/AV^−^ (Table 2). However, cEVs enriched with CD31 (PECAM-1) marker may originate either from platelets or the endothelium.

**Figure 3:**
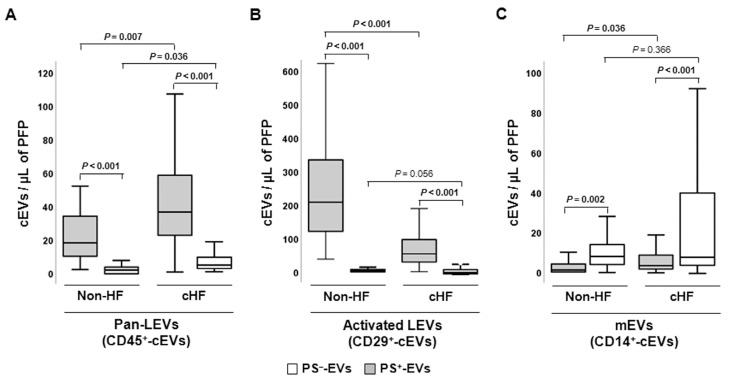
Distribution of leukocyte-derived circulating extracellular vesicles in reference non-heart failure and chronic heart failure groups. Box and whisker plots show numbers of cEVs per microliter of platelet-free plasma (cEVs/µL of PFP) of (**A**) leukocyte-derived (CD45^+^), (**B**) activated leukocyte-derived (CD29^+^), and (**C**) monocyte-derived (CD14^+^) circulating extracellular vesicles in non-heart failure control subjects (*n* = 21) and chronic heart failure patients (*n* = 119). Lines within boxes represent median values, the upper and lower boxes represent the 25th and 75th percentiles, respectively, and the upper and lower boxes outside the boxes represent the 10th and 90th percentiles, respectively. A *p* < 0.05 was considered significant (Mann–Whitney U test). *p*-values in bold correspond to significant differences. CD indicates cluster of differentiation; cHF, chronic heart failure; cEVs, circulating extracellular microvesicles; HF, heart failure; LEVs, leukocyte-derived EVs; mEVs, monocyte-derived EVs; PFP, platelet-free plasma; PS^+^, EVs exposing phosphatidylserine; and PS^−^-, EVs that do not expose phosphatidylserine.

Leukocyte-derived EVs (LEVs) carrying pan-leukocyte CD45^+^ (LCA) antigen and CD29^+^ (β1 integrin), a marker of activated leucocytes, were found to be enriched in PS^+^-EVs, whereas monocyte CD14^+^ (LPS-receptor) epitope was found to be enriched in the PS^−^-EVs population both in the non-HF individuals and cHF patients (*p* ≤ 0.002, all) (Figure 3).

Regarding distinct subtypes of inflammatory cell-derived EVs, cHF patients were characterised by displaying CD11b^+^ (macrophage-1 antigen (MAC-1), a marker of activated leukocytes) and CD15^+^ (Sialyl Lewis X, a marker of neutrophils), as well as CD3^+^ (T-cell co-receptor, a lymphocyte marker) and CD56^+^ (neural cell adhesion molecule-1 (NCAM1), a Natural Killer (NK) cell marker) epitopes in both PS^+^-EV and PS^−^-EV pools. Thus, CD11b^+^/AV^+^-LEVs and CD15^+^/AV^+^-nEVs were significantly increased compared to CD11b^+^/AV^−^-LEVs and CD15^+^/AV^−^-nEVs, respectively (*p* < 0.001, both). The contrary occurred with the numbers of CD3^+^/AV^+^-ℓEVs and CD56^+^/AV^+^-NKc-EVs, which were reduced compared to CD3^+^/AV^−^-LEVs and CD56^+^/AV^−^-nEVs, respectively (*p* < 0.001, NKc-EVs), as shown in Figure S1.

Concerning the non-HF reference group, no differences between the PS^+^-EVs and PS^−^-EVs carrying CD11b^+^, CD15^+^, CD3^+^, and CD56^+^ surface molecules were observed in the non-HF subjects (*p* > 0.194, all). Indeed, both the PS^+^-EVs and PS^−^-EVs observed in the reference non-HF group had a negligible content of CD3^+^ antigen (Figure S1).

In general, subsets of specific LEVs were observed in lower levels in the non-HF subjects compared to the patients with cHF, independently of their PS exposure. Thus, significantly reduced levels of PS^+^-EVs carrying CD45^+^ (LEVs; *p* = 0.007), CD11b^+^ (LEVs; *p* < 0.001), CD14^+^ (mEVs; *p* = 0.036), and CD15^+^ (nEVs; *p* = 0.001) markers were observed in the non-HF subjects in relation with the patients with cHF. On the contrary, CD29^+^/AV^+^-EVs (LEVs; *p* < 0.001) were increased in non-HF controls compared to patients with cHF. Regarding PS^−^-EVs, significantly reduced levels of CD3^+^/AV^−^-EVs (ℓEVs; *p* = 0.003), CD45^+^/AV^−^-EVs (LEVs; *p* = 0.0036), CD15^+^/AV^−^-EVs (nEVs; *p* < 0.001), and CD56^+^/AV^−^-EVs (NKc-EVs; *p* = 0.005) were found in the non-HF control subjects compared to the cHF patients (Figure S1).

## 3. Discussion

**Figure 4:**
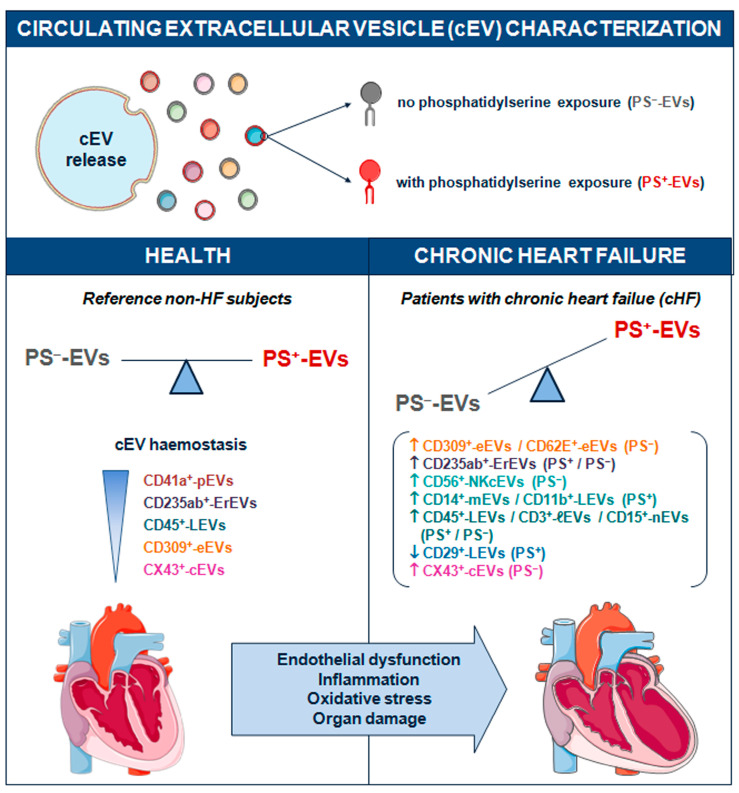
Schematic summary showing main study results of the circulating extracellular vesicle characterization focusing on phosphatidylserine membrane exposure in chronic heart failure. CD indicates cluster of differentiation; cEVs, circulating extracellular vesicles; cHF, chronic heart failure; CX43, connexin 43; eEVs, endothelial cell-derived EVs; ErEVs, erythrocyte-derived EVs; EVs, extracellular microvesicles; ℓEVs, lymphocyte-derived EVs; LEVs, platelet-derived EVs; nEVs, neutrophil-derived EVs; NKcEVs, NK cell-derived EVs; mEVs, monocyte-derived EVs; PFP, platelet-free plasma; PS^+^, EVs exposing phosphatidylserine; and PS^−^, EVs that do not expose phosphatidylserine.

No specific generic marker binding all circulating EVs has been found yet. Until now, the surface phosphatidylserine content in EV membranes detected by annexin V labelling has been widely used as a universal reporter of cEVs. However, it is now well-recognised that EVs from different cell lineages can be released either with or without the externalization of this phospholipid in their membranes [7,8,9,10,11,29]. PS^−^-EVs have been described in several distinct pathologies including multi organ dysfunction syndrome (MODS) and sepsis [30], systemic lupus erythematous (SLE) [31,32], antiphospholipid syndrome (APS) [33,34], type 1 diabetes mellitus [35], ischaemic stroke (IS) and transient ischaemic attack (TIA) [36], familial hypercholesterolemia [11,37], sickle cell disease [38], gastric cancer [39], carotid artery stenting [40], and ageing [41]. The literature regarding all-type cEVs in cHF is generally scarce [19,28,42,43] and there is no information on PS^−^-EVs. We have previously studied the role of PS^+^-EVs released by platelets [12] and immunity cells [26] in cHF patients. Here, we have focused on the cardiovascular system-related (heart, blood, and vascular resident cells) circulating PS^−^-EVs from patients with chronic heart failure, unravelling that endothelial and red blood cells are stressed in the context of cHF, as illustrated by high cEV shedding (Figure 4).

In agreement with other studies [33,34], we found that cHF is also characterised by displaying a higher quantity of EVs that do not expose PS in their membranes (PS^−^-EVs). Regarding the specific parental cell origin of cEVs, we have observed differential patterns of PS^+^-cEVs and PS^−^-cEVs in controls and in patients with cHF. Firstly, even though both groups presented higher levels of endothelium-derived cEVs that did not expose PS, cHF patients presented increased levels of PS^−^-cEVs compared to controls, supporting the notion of endothelial cell injury as a trigger of eEV release, in a similar fashion as in other conditions [44]. In fact, these results are also in agreement with Nozaki et al., who reported increased levels of cEVs presenting the endothelial cell marker CD144^+^ (VE-cadherin) but did not evaluate their PS exposure in cHF patients [19]. Thus, increased levels of eEVs might reflect the endothelial dysfunction present in cHF [45].

Despite there being controversial results [7,8,9,10,11,12], we have also confirmed that platelet-derived cEVs are mainly PS^+^ in cHF patients, as we [12] and others [46] have previously described. It is interesting to highlight the role of CD31^+^-cEVs that are not exclusively platelet-derived and could also be shed by endothelial and white blood cells. The behaviours of CD31^+^/AV^+^-cEVs and CD31^+^/AV^−^-cEVs are completely different, the former following the trend of pEVs (decreased in cHF) and the later following the trend of eEVs (increased in cHF). In light of these data, the two EV subtypes could be considered as two independent populations. Indeed, other authors have already pointed out a differential pathophysiological role of EVs depending on their PS exposure profile. In a study comprising patients that had suffered an IS or TIA, CD41^+^/PS^−^, CD62P^+^/PS^−^, and CD142^+^/PS^−^ were associated with an increased risk of the primary outcome (considered as fatal or non-fatal myocardial infarction and/or fatal or non-fatal recurrent ischaemic stroke), while CD41^+^/PS^+^, CD62P^+^/PS^+^, and CD142^+^/PS^+^ were associated with a decreased risk [36].

The population of cEVs released from inflammatory cells showed the highest degree of complexity. The levels of specific PS^−^-LEVs derived from pan-leukocytes (CD45^+^/AV^−^-LEVs), lymphocytes (CD3^+^/AV^−^-ℓEVs), neutrophils (CD15^+^/AV^−^), and NK cells (CD56^+^/AV^−^-NKcEVs) were found to be elevated as compared to controls. Similarly to PS^+^-LEVs, PS^−^-LEVs could reflect the non-resolving inflammatory state characteristic of cHF patients [47,48]. In contrast to what we have observed with pEVs and eEVs, different circulating LEV subsets present a variable pattern in terms of PS membrane externalization according to their cellular origin and activation status. The balance of PS exposure of each LEV subtype (PS^+^-LEV/PS^−^-LEV) might be an indicator reflecting their heterogeneous role in chronic heart failure.

Another class of cEVs that is often overlooked is those of red blood cell (RBC) origin [49], which are important players in the development of chronic heart failure due to their prothrombotic and pro-oxidative effects. There were no differences between erythrocyte-derived PS^+^-EVs and PS^−^-EVs levels in the blood of controls, and both subtypes were significantly increased in the blood of cHF patients, with PS^+^-EVs representing the highest number of ErEVs. Interestingly, Shet et al. observed increased numbers of PS^+^-EVs ErEVs in sickle cell disease in comparison with controls, but, when considering their PS exposure, found similar levels between PS^+^-EVs and PS^−^-EVs ErEVs [38]. On the contrary, increased levels of PS^−^-EVs ErEVs were observed in a study comprising patients with MODS and sepsis compared to controls, while the levels of PS^+^-EVs ErEVs were similar [30]. Altogether, these studies demonstrate that RBC-derived EVs display an opposite profile in terms of phosphatidylserine externalization and suggest that the ratio of PS-exposing ErEVs seems to be context- and disease-specific. Thereby, what type of erythrocyte-originated EVs (PS^+^-ErEVs/PS^−^-ErEVs) associate to vascular dysfunction in chronic heart failure deserves further attention.

Connexin-43 (CX43), a widely distributed tissue interstitial gap junction as well as mitochondrial protein, is secreted in extracellular vesicles and participates in cell communication processes. To our knowledge, the levels of EVs presenting CX43 have not been analysed before, and neither has it been investigated whether this marker is co-expressed with PS in chronic heart failure patients. It is well-established that alterations in gap junctions and in the expression of connexins occur in human heart diseases including heart failure, connexins being emerging therapeutic targets. Albeit being found across organs and tissues, CX43 is predominantly detected in endothelial, epithelial, and cardiac cells. Regarding the latter, CX43 is the main connexin form in the heart and is abundantly expressed in the ventricular myocardium. Thus, we have explored CX43 as a surrogate measure of tissue and cell damage state. Interestingly, the majority of CX43^+^-cEVs were PS^−^-cEVs in both studied populations. High levels of PS^−^-CX43^+^-EVs in cHF patients add evidence regarding the myocardial stress and injury frequently observed in cHF.

Phosphatidylserine is not only a marker of apoptosis but also of other cell death mechanisms [50]. On the other hand, the biogenesis of those cEVs that do not expose PS in their membrane is still unknown. Several theories have emerged in the last few years, including that PS^−^-EVs are true PS^+^-EVs with (1) very low PS exposure or (2) PS-masking (through endogenous lactadherin or β_2_ glycoprotein 1 (β2GP1) binding) [33,35,36]. The latter could either hamper proper PS^+^-EVs recognition or block PS-mediated EV clearance [33,34,36]. A previous work studying the clearance ratio of PS-exposing vesicles in immune mice (with high levels of the antiphospholipid antibody β_2_GP1) showed that PS^−^-EVs were cleared significantly slower than their PS-exposing counterparts [51]. This hypothesis was further supported by the findings of two studies in SLE and APS, where PS^−^-EVs were the main type of EVs found in patients but not in controls [31,34]. Similarly, it has been hypothesized that PS^−^-EVs could circulate in blood with a long half-life due to less uptake by liver macrophages [52]. Nevertheless, further research on the biological role of PS^−^-EV shedding is warranted. An increasing number of studies with patients that do not present antiphospholipid antibodies preventing EV clearance or detection have revealed high levels of PS^—^EVs, such as in type-1 diabetic patients and elderly individuals [35,41], while patients suffering from IS or TIA [36] and atrial fibrillation [53] exhibited greater amounts of PS^+^-EVs.

Furthermore, the pathophysiological role exerted by PS^−^-EVs also remains unclear. The presence of PS in the EV membranes might likely discriminate two populations with different roles, with PS^+^-EVs suggested to be more procoagulant and PS^−^-EVs reflecting a proinflammatory state [8,41]. The potential procoagulant proprieties of PS^+^-EVs are widely accepted and have already been demonstrated in several studies [54,55]. In heart failure conditions, Kou et al. determined that the exposure of PS in both blood cells and cEVs is related to the procoagulant state of these patients [28,56]. As in the inflammatory role of PS^−^-EV, high levels of PS^−^-EVs have been detected in inflammatory diseases such as SLE or APS [31,34]. Further, cHF patients present a chronic and unresolved inflammatory state, which could also be reflected in the high proportion of PS^−^-EV release. In addition, increased levels of inflammation are observed in elderly individuals in comparison with younger subjects [57], who were already shown to display higher proportions of PS^−^-EVs [41].

Although the study of EV populations without PS exposure is still in its infancy, it is evident that their release and function is unrelated to those EVs bearing PS. Our clinical model of study involving a chronic heart disease such as heart failure, which is a condition with a large systemic affectation and cellular stresses, compared with non-HF control subjects has evidenced the presence in blood of different types of circulating EVs. While immune cells secrete a variety of cEVs depending on cell type and red blood cells, both PS^−^ and PS^+^-EVs, those released by platelets are mainly PS^+^-EVs and those released by endothelial cells and connexin 43^+^-rich cells are mainly PS^−^-EVs. These differences should be taken into consideration in future studies addressing the role of cEVs in cHF.

## 4. Conclusions

In conclusion, our findings shed new light on the link between cellular stresses, endothelial dysfunction, pro-oxidant and pro-inflammatory environments, and organ damage in the context of chronic heart failure, as uncovered by a high degree of distinct cell activation processes leading to extracellular vesicle shedding in subjects’ circulation. Thus, unveiling the cEV-related complexity (in terms of phosphatidylserine externalization and parental cell origin and activation status) might help to better understand pathological cell behaviour and the intricate cellular crosstalk of chronic heart failure.

## 5. Materials and Methods

### 5.1. Healthy and Diseased Blood Donors

Guideline-directed medically treated chronic heart failure patients from the outpatient HF unit of our hospital were enrolled for this study, including patients with both preserved left ventricular ejection fraction (LVEF) (≥50%) and reduced LVEF (<40%). As a reference to cHF patient group, a non-heart failure control group (for circulating extracellular microvesicle values) was matched by age (65.7 ± 8.6 years (non-HF) and 67.0 ± 11.8 years (cHF)) and sex (24% (non-HF) and 32% (cHF) women). Individuals with HF with mild reduction in ejection fraction (40–50% of LVEF), previous inflammatory disorders, infections, sepsis, or cancer, as well as pregnant women, were excluded from the study. Baseline demographic and pharmacological data of all individuals (cHF patients and reference non-HF subjects) recruited for the study are listed in Table 3 and Table S1, respectively.

The ethics committee for clinical research of our institution, Hospital de la Santa Creu i Sant Pau in Barcelona (Spain), approved the study protocol (Reference16/44), which was conducted according the Declaration of Helsinki. Before recruitment, written informed consent was obtained from all participants.

### 5.2. Blood Sampling and Circulating Extracellular Vesicle Isolation

Venous blood was withdrawn from the cubital vein without tourniquet using a 20-gauge needle after 10–14 h of fasting into 3.8% sodium citrate tubes (Vacutainer, Becton Dickinson (BD)). After the extraction and within the first 2 h, samples were identically processed to isolate platelet-free plasma (PFP) by a two-step centrifugation protocol [26,58,59] as follows: firstly, blood cells were removed by low speed centrifugation at 1560× *g* for 20 min at 20 °C (Eppendorf 5810R GLOOB04932 centrifuge, A-4-81 rotor, Eppendorf, Hamburg, Germany) to avoid in vitro platelet activation. Next, platelet-poor plasma (PPP) was carefully aspirated, leaving about a 1 mm undisturbed layer on top of cells. Then, a second identical centrifugation was pursued at 1560× *g* for 20 min at 20 °C (Eppendorf 5415R centrifuge, FA45-24-11 rotor, Eppendorf) to obtain PFP by ensuring a complete removal of cells. Finally, PFP aliquots were stored at −80 °C until flow cytometry studies.

A two-step high-speed centrifugation was used to isolate cEVs from PFP, as previously described [26,58,59]. In brief, thawed PFP was centrifuged at 1500× *g* for 10 min at 20 °C (5417R centrifuge, FA45-24-11 rotor, Eppendorf). Then, a volume of 250 µL of PFP from the upper part of the vial was carefully transferred to a new tube. This tube was then centrifuged at 20,000× *g* for 30 min at 20 °C to pellet the cEVs. The supernatant was discarded and the cEV-enriched pellets were washed with citrate-phosphate buffered saline (PBS) solution (citrate-PBS; 1.4 mmol/L phosphate, 154 mmol/L NaCl, 10.9 mmol/L trisodium citrate, pH 7.4) before an identical centrifugation was pursued. Finally, the remaining pellets were resuspended in a final volume of 100 µL citrate-PBS. Citrate-PBS was filtered through 0.22 μm pores before each analysis on a daily basis. The procedure for flow cytometric analysis of circulating EVs followed the MIFlowCyt-EV and MIFlowCyt checklists, as previously reported [12,26].

### 5.3. Flow Cytometric Analysis of Circulating Extracellular Vesicles

Three-label flow cytometric analysis was pursued as previously described [26,58,59]. In short, washed cEV suspensions were diluted in PBS containing 2.5 mmol of CaCl_2_ (annexin binding buffer [ABB], BD Biosciences, San Jose, CA, USA). Next, combinations of AV conjugated with CF-Blue (to detect PS) and two monoclonal antibodies (mAb) labelled with phycoerythrin (PE) or fluorescein isothiocyanate (FITC) were added and incubated for 20 min at 20 °C in the dark (Table S2). Samples were then diluted with ABB and immediately analysed on a FACSCantoII™ (BD, Franklin Lakes, NJ, USA) flow cytometer.

Each sample was acquired for 1 min at *low flow* rate. Forward scatter (FSC), side scatter (SSC), and fluorescence data were obtained with the settings in the logarithmic scale. Gate limits were established following previously described criteria [26,58,59]. Megamix-Plus FSC beads (BioCytex, Marseille, France) were used to set the upper threshold for FSC. These beads are a mix of beads with diameters of 0.1 µm, 0.3 µm, 0.5 µm, and 0.9 µm, and according to their signal, the lower detection limit was set as a threshold above the electronic background noise of the flow cytometer for FSC and the second logarithm for SSC. EVs within the established gate limits (>0.1 to 1 µm) were identified and quantified based on their reactivity to cell-specific mAb and binding to AV. To identify positive marked events, thresholds of fluorescence were also set based on samples incubated with the same final concentration of isotype-matched control mAb after titration experiments. AV binding level was corrected for auto-fluorescence using fluorescence signals obtained with EVs in a calcium-free buffer (PBS). Additional controls to correct for PE-, FITC-, and CFBlue-fluorescence were also performed (unstained and single-stained controls), as well as serial dilutions to ensure proper event detection and swarming prevention. Finally, to confirm the presence of EVs in the cEVs suspension, 5% saponin-treated controls were investigated. Buffers were prepared daily and filtered through 0.22 µm pore-size filters under vacuum to reduce background noise.

BD FACSDiva™ Software (version 6.1.3, Becton Dickinson, Franklin Lakes, NJ, USA) was used to analyse data. Nieuwland’s formula [60] was used to calculate cEV concentration (number of EVs per µL of platelet-free plasma). Considering sample’s volume, flow cytometer’s flow rate, and number of fluorescence-positive events (N), the formula is as follows: EVs/µL = N × (Vf/Va) × (Vt/FR) × (1/Vi), where Vf(µL) = final volume of washed EVs suspension, Va(µL) = volume of washed EVs suspension used for each labelling analysis, Vt(µL) = volume of EVs suspension before fluorescence-activated cell sorting analysis, FR(µL/min) = flow rate of the cytometer at low mode (the average volume of cEV suspension analysed in 1 min), 1 is the microliter unit of volume, and Vi(µL) = original volume of plasma used for cEVs isolation.

To calculate which cEVs did not have PS in their membrane, the number of AV^+^ events (PS^+^) was subtracted from the total number of fluorescence-positive gated events (which included PS^+^ and PS^−^ events), obtaining the number of PS^−^-EVs. Once obtained, this value was used to calculate EVs concentration by using the formula stated above [60].

### 5.4. Statistical Analysis

Statistical analyses were pursued using SPSS Statistical Analysis System (version 26.0, IBM Corp. Armonk, NY, USA). Shapiro–Wilk test was utilised to assess variables’ normality. Descriptive analyses for quantitative variables were expressed using mean ± standard deviation or median (interquartile range 0.25–0.75), whereas, for qualitative variables, the number of cases and percentages were utilised. Chi-squared analysis was used to compare the frequencies of qualitative variables between groups. Non-parametric tests were used to contrast median values of quantitative variables and a *p* < 0.05 was considered statistically significant. Sample size was determined using the GRANMO sample size calculator (version 7.12, April 2012). To detect mean differences in the number of cEVs, a total of 126 individuals (healthy and diseased subjects) would be needed to complete the study (α risk = 0.05, beta risk = 0.2, two-sided test).

## Figures and Tables

**Table 1:** Absolute numbers and relative amounts of annexin V-positive and -negative circulating extracellular vesicles in reference to non-heart failure subjects and chronic heart failure patients.

cEVs		cHF (*n* = 119)	Non-HF (*n* = 21)	*p*-Value
**AV^+^-EVs + AV** **^−^-EVs**		1079.12 [425.93–4702.2]	803.72 [300.6–1104.67]	0.135
**AV^+^-EVs**				
**Total**	n	159.6 [110.8–252.37]	309.2 [121.14–453.31]	**0.022**
	%	15.76 [3.76–32.47]	51.48 [28.18–78.19]	**<0.001**
**CD309^+^**	n	1.9 [0–7.52]	0 [0–2.90]	0.154
	%	0.61 [0–2.16]	0 [0–0.45]	0.094
**CD41a^+^**	n	124 [76.1–180.5]	406 [110–646]	**0.001**
	%	53.4 [44.03–62.52]	85.61 [77.11–96.76]	**<0.001**
**CD45^+^**	n	36 [21.45–58]	18 [9–38]	**0.007**
	%	16.49 [11.37–24.59]	9.01 [2.24–18.77]	**0.016**
**CD235ab^+^**	n	49.7 [17.5–88.35]	4.3 [1.75–8.98]	**<0.001**
	%	24.17 [13.91–33.10]	1.33 [0.54–2.49]	**<0.001**
**CX43^+^**	n	4.6 [2–7.98]	1.6 [0.8–8.0]	0.083
	%	1.88 [1.05–2.98]	0.56 [0.17–1.65]	**0.001**
**AV^−^-EVs**				
**Total**	n	787.8 [262.2–4576.7]	253.86 [99.57–703.16]	**0.007**
	%	84.25 [67.53–96.24]	48.52 [21.80–71.82]	**<0.001**
**CD309^+^**	n	12 [4–43.32]	2 [1–8.44]	**<0.001**
	%	23.08 [10.4–41.24]	12.82 [2.03–19.05]	**0.012**
**CD41a^+^**	n	4.16 [2.0–9.5]	5 [0.5–14.17]	0.773
	%	6.76 [1.15–13.89]	20.41 [0–38.9]	**0.032**
**CD45^+^**	n	4 [1.92–9.12]	2.2 [0–4.0]	**0.036**
	%	5.81 [1.74–12.51]	6.01 [0–14.28]	0.586
**CD235ab^+^**	n	14 [4.63–28.5]	6.0 [4.0–11.0]	**0.015**
	%	19.56 [9.1–36.49]	27.4 [11.11–47.62]	0.245
**CX43^+^**	n	18.60 [5.4–43.2]	4.4 [1.6–14.0]	**0.001**
	%	27.2 [15.23–42.81]	18.06 [9.09–30.43]	**0.028**

Data are expressed as median (interquartile range) of absolute values and relative expression. AV^+/^^−^ indicates annexin V^+/−^; CD, cluster of differentiation; cEVs, circulating extracellular vesicles; cHF, chronic heart failure; EVs, extracellular vesicles; and HF, heart failure.

**Table 2:** Absolute numbers of distinct subsets of platelet-derived annexin V-positive and negative circulating extracellular vesicles in reference non-heart failure subjects and chronic heart failure patients.

pEVs	cHF Patients	Non-HF Subjects	*p*-Value
**AV^+^-pEVs**			
**CD31^+^**	28.5 [16–58.9]	166 [53–283.78]	**<0.001**
**CD41a^+^**	124 [80–178.6]	406 [126–642]	**0.001**
**CD31^+^/CD41a^+^**	28.25 [12–58.9]	122 [40–220]	**0.001**
**CD62P^+^**	30 [14–51.3]	58 [31–92]	**0.010**
**AV** **^−^-pEVs**			
**CD31^+^**	17.1 [6–44]	4 [2–10]	**0.002**
**CD41a^+^**	4.16 [2–9.5]	5 [1–12.78]	0.773
**CD31^+^/CD41a^+^**	0 [0–0]	0 [0–0]	0.123
**CD62P^+^**	2 [0–6]	0 [0–4]	0.085

Data are expressed as median (interquartile range) of absolute values. AV^+/^^−^ indicates annexin V^+/−^; CD, cluster of differentiation; cHF, chronic heart failure; HF, heart failure; and pEVs, platelet-derived extracellular vesicles.

**Table 3:** Clinical characteristics of the studied chronic heart failure patient population.

Parameters	Patients with cHF (*n* = 119)
**Demographic characteristics**	
Male, n (%)	81 (68)
Female, n (%)	38 (32)
Age, years	67 ± 11.8
**Clinical data**	
Systolic blood pressure, mmHg	120.4 ± 19.1
Diastolic blood pressure, mmHg	73.9 ± 11.1
Left ventricular ejection fraction, %	45.59 ± 18.98
**Comorbidities**	
Smokers, n (%)	13 (10.9)
Hypertension, n (%)	82 (68.9)
Pulmonary hypertension, n (%)	49 (41.1)
Dyslipidaemia, n (%)	64 (53.7)
Chronic kidney disease, n (%)	46 (38.6)
Diabetes mellitus, n (%)	53 (44.5)
Atrial fibrillation, n (%)	50 (42)
**Background medication**	
Angiotensin-converting-enzyme inhibitors, n (%)	48 (40.3)
Angiotensin II receptor blockers, n (%)	35 (29.4)
Angiotensin receptor neprilysin inhibitors, n (%)	17 (14.2)
Beta-blockers, n (%)	100 (84)
Aldosterone antagonists, n (%)	66 (55.4)
Diuretics ^a^, n (%)	103 (86.5)
Ivabradine, n (%)	14 (11.7)
Statins, n (%)	77 (64.7)
Insulin, n (%)	16 (13.4)
Anti-diabetic drugs, n (%)	40 (33.6)
Anticoagulants, n (%)	61 (51.2)
Antiplatelet agents, n (%)	46 (38.6)
Anti-arrhythmic drugs, n (%)	26 (21.8)

Data are expressed either by mean ± standard deviation or number of cases (percentage). ^a^ Diuretics include furosemide, hydrochlorothiazide, torasemide, and indapamide. **cHF** indicates chronic heart failure.

## Data Availability

Any data used for this manuscript can be obtained from the authors upon reasonable request.

## Supplementary Materials

The following supporting information can be downloaded at: https://www.mdpi.com/article/10.3390/ijms241411824/s1.
