# Supplementary material for: Red Blood Cells and Endothelium Derived Circulating Extracellular Vesicles in Health and Chronic Heart Failure: A Focus on Phosphatidylserine Dynamics in Vesiculation
Source: Int J Mol Sci. 2023 Jul 23;24(14):11824. doi: 10.3390/ijms241411824 (PMC10380787; doi:10.3390/ijms241411824)
Supplement: Supplementary file 1 [file ijms-24-11824-s001.zip › ijms-2500470-supplementary.pdf]

SUPPLEMENTARY MATERIAL

Red blood cells and endothelium derived circulating extracellular vesicles in health and chronic heart failure: a focus on phosphatidylserine dynamics in vesiculation

Rosa Suades,[1,2] Alba Vilella-Figuerola,[1] Teresa Padró,[1,2] Sonia Mirabet,[2,3*] Lina Badimon[1,3,4*]

[1] Cardiovascular Program ICCC, Research Institute of Hospital Santa Creu i Sant Pau, IIB Sant Pau, Barcelona, Spain; [2] Centro de Investigación Biomédica en Red Cardiovascular (CIBER-CV), Instituto de Salud Carlos III, Madrid, Spain; [3] Heart Failure Group, Cardiology Department, Hospital Santa Creu i Sant Pau, Barcelona, Spain; [4] Cardiovascular Research Chair, Universitat Autònoma de Barcelona (UAB), Barcelona, Spain

*Both authors contributed equally

Address for corresponding author:

Prof. Lina Badimon
Cardiovascular-Program ICCC
Research Institute Hospital Santa Creu i Sant Pau
Sant Antoni Mª Claret 167, 08025 Barcelona, Spain
Phone: +34.935565882
Fax: +34.935565559
E-mail: lbadimon@santpau.cat

Authors' ORCID

RS: 0000-0002-0193-3115
AV-F: 0000-0001-6957-4043
TP: 0000-0003-1921-954X
SM: 0000-0001-5955-2748
LB: 0000-0002-9162-2459

SUPPLEMENTAL FIGURE

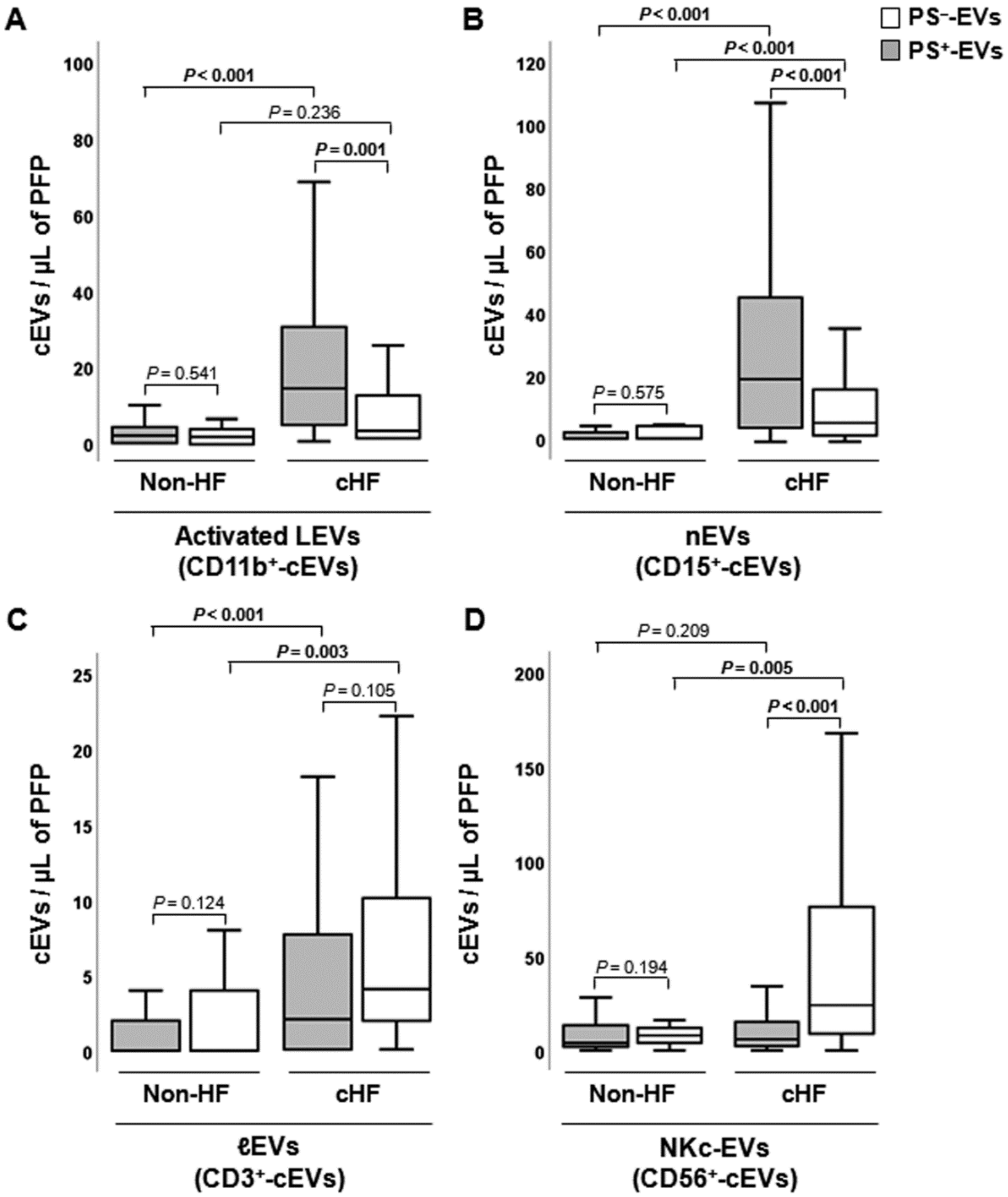

Figure S1: Distribution of leukocyte-derived circulating extracellular vesicles in a group of chronic heart failure patients and a reference non-heart failure group. Box and whisker plots show numbers of EVs per microliter of platelet-free plasma (EVs/µL of PFP) from (**a**) activated leukocytes ($CD11b^+$), (**b**) neutrophils ($CD15^+$-nEVs), (**c**) lymphocytes ($CD3^+$-ℓEVs), and (**d**) NK cells ($CD56^+$NKc-EVs) according to their PS membrane exposure in chronic heart failure patients (n=119) and a reference non-heart failure subjects (n=21). Lines within boxes represent median values, the upper and lower boxes represent the 25th and 75th percentiles, respectively, and the upper and lower boxes outside the boxes represent the 10th and 90th percentiles, respectively. A $P < 0.05$ was considered significant (U-Mann Whitney test). P-values in bold correspond to significant differences. **cHF** indicates chronic heart failure; **EVs**, extracellular vesicles; **HF**, heart failure; **ℓEVs**, lymphocyte-derived EVs; **LEVs**, leukocyte-derived EVs; **nEVs**, neutrophil-derived EVs; **NKcEVs**, NK cell-derived EVs; **PFP**, platelet-free plasma; **PS^+**, EVs exposing phosphatidylserine; and **PS^{-}**: EVs that do not expose phosphatidylserine.

SUPPLEMENTARY TABLES

Table S1. Clinical characteristics of the studied reference non-heart failure group.

	Reference non-HF subjects (n=21)
**Demographic characteristics**	
Male, n (%)	16 (76)
Female, n (%)	5 (24)
Age, years	65.7 ± 8.6
**Clinical data**	
Systolic blood pressure, mmHg	145.2 ± 24.2
Diastolic blood pressure, mmHg	84.8 ± 14.2
Left ventricular ejection fraction , %	52-74 [a]
**Comorbidities**	
Smokers, n (%)	2 (9.5)
Hypertension, n (%)	11 (52.3)
Pulmonary hypertension, n (%)	-
Dyslipidaemia, n (%)	14 (66.6)
Chronic kidney disease, n (%)	1 (4.7)
Diabetes mellitus, n (%)	3 (14.2)
Atrial fibrillation, n (%)	-
**Background medication**	
Angiotensin-converting-enzyme inhibitors, n (%)	7 (33.3)
Angiotensin II receptor blockers, n (%)	3 (14.2)
Angiotensin receptor neprilysin inhibitors, n (%)	-
Beta-blockers, n (%)	1 (4.7)
Aldosterone antagonists, n (%)	-
Diuretics, n (%)	1 (4.7)
Ivabradine, n (%)	-
Statins, n (%)	13 (61.9)
Insulin, n (%)	1 (4.7)
Anti-diabetic drugs, n (%)	2 (9.5)
Anticoagulants, n (%)	1 (4.7)
Antiplatelet agents, n (%)	5 (23.8)
Anti-arrhythmic drugs, n (%)	-

Data are expressed either by mean ± standard deviation or number of cases (percentage). **HF** indicates heart failure. [a]LVEF normal range (excerpt from https://www.ncbi.nlm.nih.gov/books/NBK459131/ on 25/05/2023).

Table S2: Cell surface molecules for extracellular microvesicle identification and characterisation

ANTIBODIES FOR FLOW CYTOMETRY ANALYSIS	EXPRESSION	MARKER	EPITOPE	CONJUGATION	CLONE	DILUTION	HOST	COMPANY	CATALOGUE #
**Annexin V**	Widely expressed	PS^+	PS-binding protein	CF405	-	5μl (1:10)	-	Immunostep	ANXVCFB-200T
**Endothelial cells**	Endothelial cells	$CD309^+$	VEGFR-2	PE	ES8-20E6	5μl (1:10)	Mouse	Miltenyi Biotec	130-093-598
	Activated ECs	$CD62E^+$	E-selectin	PE	68-5H11	5μl (1:100)	Mouse	BD Pharmingen	551145
**Platelets markers**	Endothelial cells, platelets	$CD31^+$	PECAM	FITC	WM59	5μl (1:100)	Mouse	BD Pharmingen	555445
	Platelets	$CD41a^+$	$\alpha_{IIb}\beta_3$-integrin	PE	HIP8	5μl (1:100)	Mouse	BD Pharmingen	555467
	Activated platelets	$CD62P^+$	P-selectin	PE	AK-4	5μl (1:100)	Mouse	BD Pharmingen	550561
**Leukocytes markers**	Lymphocytes T	$CD3^+$	T-cell coreceptor	FITC	HIT3b	5μl (1:10)	Mouse	Immunotools	21810033
	Leukocytes	$CD45^+$	LCA	PE	MEM-28	5μl (1:10)	Mouse	Immunotools	21270454
	Neutrophils, monocytes	$CD11b^+$	MAC-1	FITC	MEM-174	5μl (1:10)	Mouse	Immunotools	21279113
	Monocytes, macrophages	$CD14^+$	LPS-receptor	PE	M5E2	5μl (1:100)	Mouse	BD Pharmingen	555398
	Activated lymphocytes	$CD29^+$	ITGBI	FITC	HI29a	5μl (1:10)	Mouse	Immunotools	21810293
	Granulocytes, neutrophils	$CD15^+$	Sialyl Lewis X	PE	MEM-158	5μl (1:10)	Mouse	Immunotools	21270154
	Natural killers	$CD56^+$	NCAM1	FITC	B-A19	5μl (1:10)	Mouse	Immunotools	21810563
**Erythrocytes**	Erythrocytes	$CD253ab^+$	Glycophorin ab	FITC	HIR2	5μl (1:10)	Mouse	Immunotools	21272353
**Others**	Cardiomyocytes, cardiac cells	$CX43^+$	Connexin-43	PE	Polyclonal	1μl (1:10)	Rabbit	LifeSpan BioSciences	LS-C218833

*At staining (final volume of reagents: 50μL [5μL of isolated EVs, 5μL of Annexin V, ~5μL of antibody-FITC, ~5μL of antibody-PE, adjust to final a volume of 50μL with annexin binding buffer]).

BD indicates Becton Dickinson; **CD**, cluster of differentiation; **EC**, endothelial cells; **FITC**, fluorescein isothiocyanate; **ITGB1**, integrin β-1; **LCA**, leukocyte common antigen; **LPS**, lipopolysaccharide; **MAC-1**, macrophage-1 Antigen; **NCAM1**, neural cell adhesion molecule-1; **PE**, phycoerythrin; **PECAM**, platelet endothelial cell adhesion molecule-1; **PS**, phosphatidylserine; and **VEGFR-2**, vascular endothelial growth factor receptor 2.
